# Imidazolium-Based Ionic Liquid as Efficient Corrosion Inhibitor for AA 6061 Alloy in HCl Solution

**DOI:** 10.3390/ma13204672

**Published:** 2020-10-20

**Authors:** Xiaohong Wang, Ailing Huang, Dongquan Lin, Mohd Talha, Hao Liu, Yuanhua Lin

**Affiliations:** 1 School of New Energy and Materials, Southwest Petroleum University, Chengdu 610500, China; xhwang3368@swpu.edu.cn (X.W.); hal0731000@163.com (A.H.); ldq0613@163.com (D.L.); hao1131404542@163.com (H.L.); yhlin28@163.com (Y.L.); 2 State Key Laboratory of Oil and Gas Reservoir Geology and Exploitation, Southwest Petroleum University, Chengdu 610500, China

**Keywords:** corrosion inhibition, ionic liquid, AA 6061, XPS

## Abstract

The corrosion inhibition performance of an imidazolium-based ionic liquid (IL), 1-butyl-3-methylimidazolium thiocyanate (BMIm), was studied on AA 6061 alloy in 1 M HCl solution at 303 K, 333 K, and 363 K by gravimetric tests, potentiodynamic polarization, and electrochemical impedance spectroscopy (EIS) analysis. Scanning electron microscopy with energy dispersive X-ray (SEM-EDX) and X-ray photoelectron spectroscopy (XPS) were used to detect the surface morphologies and chemical composition of the surface films. The results indicate that this IL inhibits AA 6061 corrosion in acid with maximum inhibition efficiencies of 98.2%, 86.6%, and 41.2% obtained at 303 K, 333 K, and 363 K respectively. Inhibition efficiency generally decreased with increasing immersion time; the major exception was at 303 K, whereby the inhibition efficiency was detected to increase with immersion time from 30 to 90 min and then decrease slightly beyond 90 min. The results indicate that BMIm is a mixed-type inhibitor with a predominant effect on cathodic reactions. Surface morphology analyses by SEM revealed less surface damage in the presence of the inhibitor. XPS analysis established the development of a protective film on the AA 6061 surface which was hydrophobic in nature.

## 1. Introduction

Carbonate reservoirs require acid fracturing to improve their permeability. A section of tubing needs to be removed after acid fracturing. Steel tubing can be removed by drilling, but the efficiency is low and the cost is high. Aluminum alloys are the most promising alternative to steel tubing materials because of their high specific strength and ease of drilling through them [1]. HCl is frequently used in acid fracturing, but it can cause high corrosion rates of aluminum components [1,2]. Therefore, the key requirement for the practical application of aluminum alloy as a tubing material is to find a suitable inhibitor to reduce corrosion in acid to ensure that it can withstand the specified internal and external conditions during acid fracturing. Most effective inhibitors for Al are organic compounds that comprise heteroatoms, i.e., N, S, or O atoms, in their structure [3,4,5]. It has been shown that imidazoline-based compounds have good corrosion inhibition efficiency and ease of degradation [6]. However, many of these inhibitors are not eco-friendly. Due to health and environmental concerns, the use of several organic inhibitors has been restricted because of their toxic nature. While sol-gel coatings on aluminum alloys offer many benefits regarding chemical attack [7], but they also have limitations. Natural products from plants are also used as inhibitors and have been shown to have good inhibition efficiency [8,9,10]. However, challenges with forecasting the precise inhibiting groups, the binding sites, inhibition mechanism, etc. are significant, as the complex composition of these natural inhibitors requires separation of the extract into distinct constituents to establish these parameters. Furthermore, extracts of plants are fairly unstable and easily degraded, which limits their use on industrial scales [11].

In the meantime, ionic liquids (ILs), a kind of molten salt composed of organic cations and several anions, have found vast potential applications as favorable green inhibitors due to their properties, like good ionic conductivity, nonflammability, greater stability, low volatility at normal working temperature, and eco-friendly nature [12,13,14]. ILs display stability to thermal, electrochemical, chemical, and radiolytic perturbations [15,16]. They are usually utilized in relatively high-temperature applications. A major reason for increasing interest in ILs is their high thermal stability; they are resistant to both decomposition and evaporation [17]. Imidazolium-based ILs manifest high thermal stability [18]. Earlier workers have shown that ILs are feasible as corrosion inhibitors for various alloys in acid solutions [19,20,21]. Some ILs having N atoms in the cationic part, such as imidazolium, pyrrolidine, pyridinium, and their derivatives, have been broadly tested as inhibitors for various metals in different media [22,23,24,25]. However, to our knowledge, imidazolium-based ILs are yet to be tested as corrosion inhibitors for Al alloys. To explore this, the present work evaluates the corrosion inhibition influence exerted by 1-butyl 3-methylimidazolium thiocyanate (BMIm), an imidazolium-based IL, as a new corrosion inhibitor for AA 6061 alloy in 1 M HCl solution, as well as the effect of concentration and temperature. The corrosion inhibition property of the IL on AA 6061 alloy was determined using gravimetric analysis, the potentiodynamic polarization technique, and EIS study by adding different concentrations of ILs into corrosive solution. Surface analyses of the alloy samples were executed using SEM-EDX and XPS analysis. The hydrophobic character of samples after immersion in test solution was also evaluated using contact angle analysis. Based on the outcomes, useful facts about the mechanisms of corrosion inhibition are presented.

## 2. Experimental Details

### 2.1. Materials

Experiments were executed on AA 6061 alloy samples. The chemical composition of the alloy is presented in Table 1. AR grade 37% HCl and distilled water were as a test solution. The BMIm (98%) was obtained from Lanzhou Greenchem, Lanzhou, China, and used without further purification.

### 2.2. Gravimetric Tests

Mass loss experiments were carried out under full soaking of 250 mL of nondeaerated 1 M HCl solution with and without inhibitor at various temperatures (303 K, 333 K, and 363 K), controlled by an oil thermostat (Jintan Science Analysis Instrument Co., Ltd., Jintan, China). Aluminum alloy coupons of 25 mm × 20 mm × 2 mm with a hole of 1.5 mm diameter at one end were abraded using emery paper (Chron Chemical Co., Ltd., Chengdu, China) (grades 200, 400, 600, 800, and 1000) and then washed using distilled water and acetone. After weighing using a digital balance with an accuracy of ±0.01 mg, the samples were suspended in a beaker containing the test solution using a string. After immersion for different times, the samples were taken out, washed under running water to remove the corrosion products, dried under a hot air stream, and again weighed. Different concentrations of BMIm were used to analyze the concentration effect on the inhibition efficiency according to temperature. For accuracy, gravimetric tests were done in triplicate and the mean values are reported and used in further evaluations. Corrosion rate (*ν*) and inhibition efficiency ($\eta_{w}$%) were calculated using the equations given below [26]:

(1) $$\nu =\frac{W}{St}$$

where *W* = average weight loss of three specimens, *S* = surface area of one AA 6061 sample, and *t* = time (h) of immersion.

(2) $$\eta_{w}\%=\frac{\nu_{0}-\;\nu }{\nu_{0}}\;\times \;100$$

where *ν*_0_ and *ν* are the values obtained for corrosion rate without and with inhibitor, respectively.

### 2.3. Electrochemical Analysis

Electrochemical tests were performed using an Autolab PGSTAT302N (Metrohm AG Co., Ltd., Barendrecht, The Netherlands) with three-electrode cell association, in which a platinum wire served as a counter electrode and a dip type saturated calomel electrode (SCE) (Zhanhua electronic precision instrument service Co., Ltd., Shanghai, China) was used as the reference electrode. The AA 6061 sample (Xinjiang Joinworld Co., Ltd., Urumqi, China), which was the working electrode (WE), had an exposed surface area of 10 × 10 mm; it was abraded with emery paper on the test face, and then washed with distilled water, degreased using acetone, and finally dried. Electrochemical experiments were carried out in 1 M HCl solutions with different concentrations of BMIm (1.0, 2.0, 3.0, and 4.0 mM) at 303 K. Before measurements of all electrochemical tests, the WE was kept in the test solution for 30 min at ambient temperature to achieve fairly constant OCP values. The potentiodynamic polarization tests were performed with a potential range of −400 mV to 600 mV vs. OCP at a sweep rate of 0.5 mV s^−1^. The inhibition efficiency $(\eta_{p}\%$) was determined as:

(3) $$\eta_{p}\%=\frac{I_{\text{corr}}\;-\;I_{\text{corr}\left(\text{inh}\right)}}{I_{\mathrm{corr}}}\;\times \;100$$

EIS was performed at OCP in the frequency range of 10^5^ to 10^−2^ Hz using a 10 mV peak-to-peak voltage excitation. The impedance data were analyzed to assess the corrosion characteristics. Various parameters were obtained from the simulation of plots using the Zsimpwin 3.21 software. All the measurements were done thrice and the average values are reported using the best figures. A fresh solution was used for every electrochemical experiment.

### 2.4. Surface Analyses

Samples of dimension 10 mm × 10 mm × 2 mm were prepared as described before. After dipping in 1 M HCl without and with 3 mM inhibitor for 90 min, the samples were completely washed using distilled water and dried. SEM (ZEISS EVO MA15, Carl Zeiss AG Co., Ltd., Oberkochen, Germany) was used to visualize the morphology of the surface for the polished, corroded, and inhibited samples. Electrons were generated at the source by thermionic heating. These electrons were then accelerated to a voltage between 1–40 kV and condensed into a narrow beam applied for imaging and analysis. The equivalent EDX spectra and elemental mapping were used for the qualitative analysis of the adsorbed inhibitor on the sample surface. An X-Max SDD detector was used. XPS (Model: ESCALAB 250 XI, Thermo Fisher Scientific Co., Ltd, Waltham, MA, USA) was also used to identify the chemical composition of the films formed on the samples after immersion in 1 M HCl solution without and with 3 mM inhibitor concentration. The Al Kα line was applied as the X-ray baseline. Inspected spectra, collected with the high-resolution Al 2p, O 1s, N 1s, S 2p, C 1s, and Cl 2p regions, were verified. Nonlinear Shirley background subtraction was used to get the XPS signal intensities. A contact angle analysis of the samples was also performed using a contact angle tester with a liquid drop (DSA100 KRUSS, Bluestar Electronic Technology Co., Ltd, Shenzhen, China). The shapes of the droplets were obtained using a digital camera, and the angles were determined using a computer with the help of the JGW-360A software (Version 1.0, Chenghui Testing machine Co., Ltd. Chengdu, China). The contact angle data were obtained from the average of six recordings under identical conditions and using the best pictures.

## 3. Results and Discussion

### 3.1. Gravimetric Investigations

**Figure 1:**
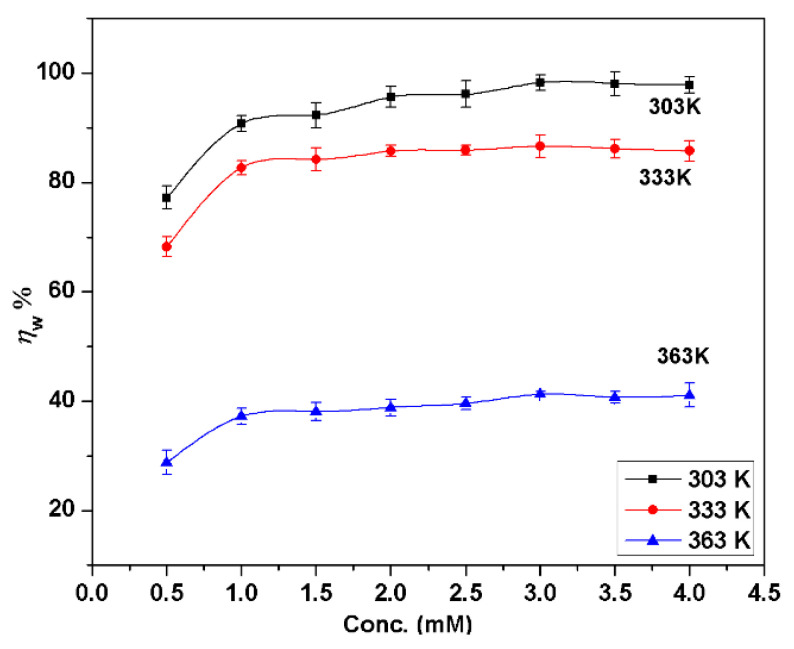
Relationship between inhibition efficiency ($\eta_{w}$) and concentration of inhibitor in 1 M HCl at different temperatures using weight loss method with immersion time of 90 min.

**Figure 2:**
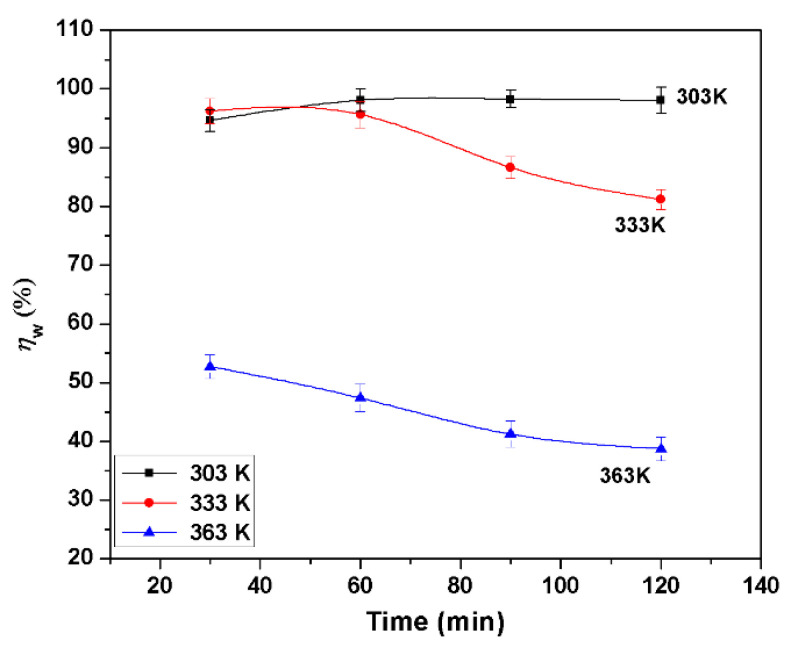
Relationship between inhibition efficiency ($\eta_{w}$) and time in 1 M HCl at different temperatures using weight loss method at 3 mM concentration of inhibitor.

The values obtained for inhibition efficiencies ($\eta_{w}$) from the mass loss (immersion time is 90 min) for different BMIm concentrations in 1 M HCl at different temperatures are shown in Figure 1. To analyze the effect of temperature on inhibition ability, tests were carried out from 303 K to 363 K in steps of 30 K. It was found that $\eta_{w}$ increased with increases in concentration at various temperatures. At 3.0 mM concentration, the values of maximum efficiencies were 98.2%, 86.6%, and 41.2% for 303 K, 333 K, and 363 K respectively. These results indicate that BMIm is a good inhibitor for Al alloy at 303 K and 333 K in 1 M HCl, but that it was not very effective at 363 K. A possible reason is that the corrosion rate was very fast at 363 K, and a large amount of aluminum had been corroded before the BMIm formed a protective film on the sample surface. In order to verify this, an additional experiment was carried out by immersing the aluminum alloy sample in deionized water containing 4.0 mM inhibitor (BMIm) for 2 h at 303 K, and then testing it in 1 M HCl solution having 3.0 mM BMIm at 363 K; the obtained $\eta_{w}$ was 71.4%. These results of supplementary experiments showed that BMIm is also an effective inhibitor at 363 K if a protective film forms on the surface before the experiment. Figure 1 also demonstrates that $\eta_{w}$ diminishes with temperature, which could be ascribed to the increased ease of inhibitor desorption from the alloy surface with temperature increase. Figure 2 shows the influence of immersion time on inhibition efficiency. Notably, $\eta_{w}$ was significantly dependent on the immersion time, increasing with the time of immersion at 303 K from 30 to 90 min, and then decreasing slightly; however, for other temperatures, it decreased regularly with an increase in immersion time. The presence of BMIm in the acid solution prevents the reduction/oxidation reactions from taking place which are responsible for metal degradation, so that an increasing inhibitor concentration enhances the adsorption of inhibitor molecules and coverage of the inhibitor on the metal surface [27].

### 3.2. Open Circuit Potential (OCP)

**Figure 3:**
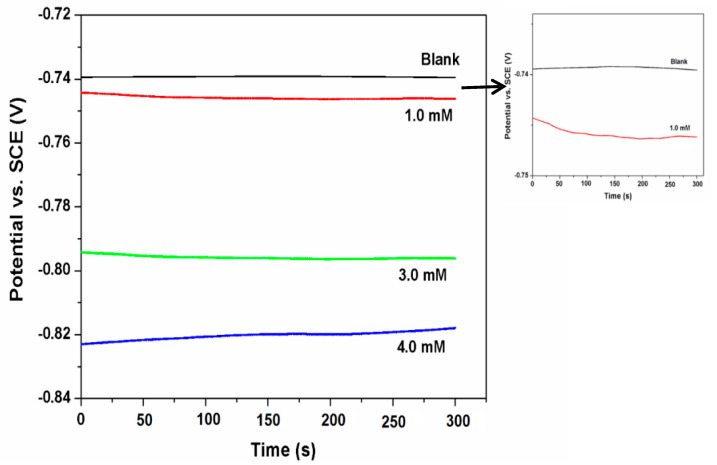
Open circuit potential versus time curves for AA 6061 in 1 M HCl without and with inhibitor at 303 K.

The variation of OCP (*E*_ocp_) of AA 6061 alloy in 1 M HCl solution with time in the absence and presence of inhibitor at 303 K is shown in Figure 3. It can be noticed from the figure that the shapes of all the curves are similar. The test specimens achieved steady-state potential in the test solution in the absence and presence of inhibitor in about 200 s, as illustrated in Figure 3. The values of potential in the presence of the inhibitor are moved towards the cathodic side, indicating the impact of the inhibitor largely on the cathodic reaction [28].

### 3.3. Potentiodynamic Polarization Study

**Figure 4:**
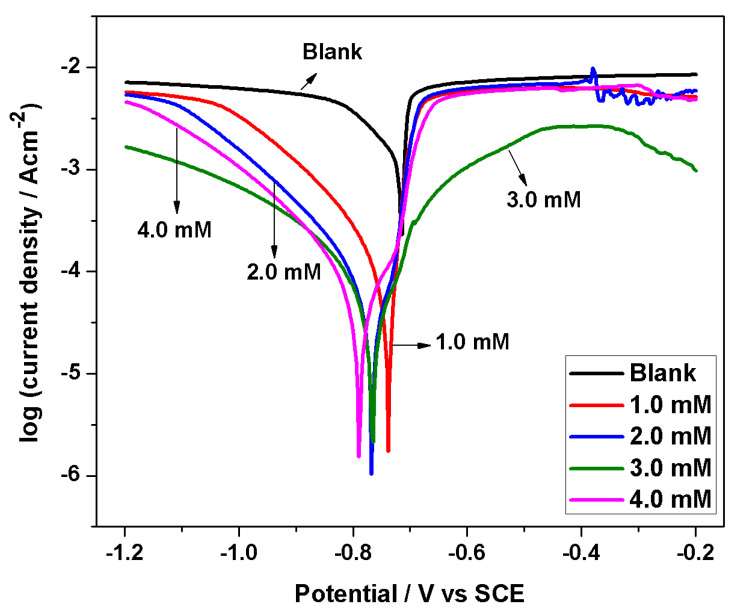
Potentiodynamic polarization curves for the corrosion of AA 6061 alloy in 1 M HCl solution without and with various inhibitor concentrations at 303 K.

The potentiodynamic polarization curves for AA 6061 alloy in 1 M HCl containing different concentrations of BMIm at 303 K with 90 min immersion time are presented in Figure 4. The corrosion current density (*I*_corr_) and other parameters were assessed by extrapolating the cathodic linear area to the corrosion potential as described before in presence of inhibitors for aluminum [26,29]. Table 2 demonstrates the various corrosion parameters obtained from polarization curves. It is clear from Figure 4 that the curves shifted towards the more negative potentials in the presence of inhibitor in comparison to the blank sample. This indicates that the inhibitor predominantly exerts an influence on cathodic reactions [30]. It appears that *I*_corr_ reduces with the concentration of the inhibitor. Likewise, efficiency increases with inhibitor concentration, which may be attributed to enhanced blockage portions of the electrode surface by adsorption of the inhibitor. The $\eta_{p}$ of a 4.0 mM inhibitor concentration reached a maximum of 98%, indicating that BMIm is a good inhibitor for AA 6061 alloy in 1 M HCl solution. It is also obvious from Table 2 that as the concentration of BMIm increases, the values of the corrosion potential (*E*_corr_) shift slightly in the negative direction. An inhibitor must be considered either anodic or cathodic when variation in the *E*_corr_ value between the inhibited and the blank sample is more than 85 mV [31]. The present results demonstrate that the differences in the *E*_corr_ values between the inhibited and the blank systems were not more than 85 mV, which suggests that the inhibitor is mixed-type, with a predominant influence on cathodic reactions [32,33]. Cathodic slope alters to some extent upon the addition of inhibitor, which indicates that the mechanism of hydrogen evolution is not significantly changed in the presence of the inhibitor.

### 3.4. Electrochemical Impedance Spectroscopy (EIS) Analysis

**Figure 5:**
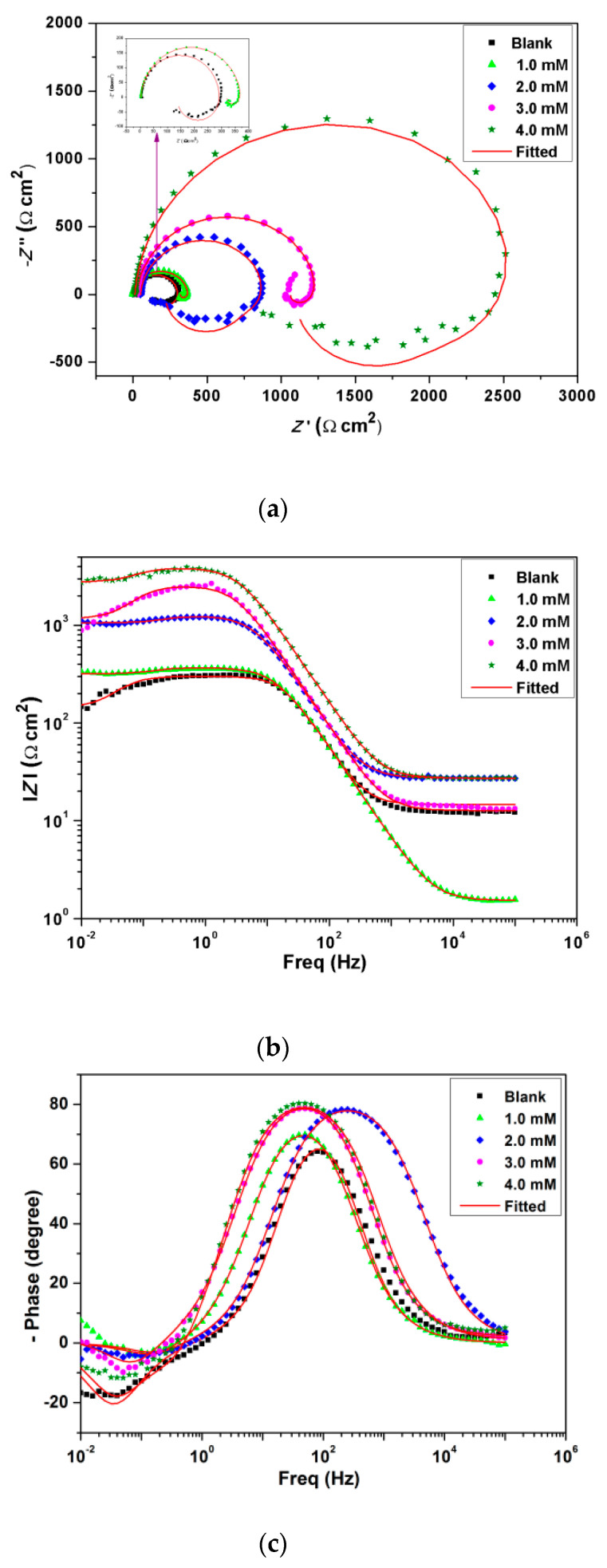
Impedance plots of the of AA 6061 corrosion in 1 M HCl without and with different concentrations of inhibitor at 303 K, (**a**) Nyquist Plots, (**b**) Bode modulus plots, (**c**) Bode phase plots.

To get information about the surface passive films on the aluminum alloy samples, an EIS investigation in 1 M HCl without and with different concentrations of inhibitor was executed (immersion time is 90 min). Figure 5a shows the corresponding Nyquist diagram, and Figure 5b,c the Bode diagrams of the investigated samples at 303 K. The Nyquist plots present a high frequency (HF) capacitive loop and a low frequency (LF) inductive loop. Comparable plots have also been reported by other investigators for the corrosion of aluminum and its alloys in acidic media [34,35]. The HF capacitive loop could be ascribed to the charge transfer resistance of the oxide layer on Al, and the LF loop to the process of H^+^ ion relaxation and the adsorption of corrosive ions (mainly anions), i.e., chloride on or into the oxide film [36,37,38]. An inductive loop was also ascribed to the dissolution of Al at low frequencies or the redissolution of the surface oxide layer [39,40]. Inductive behavior can be attributed to surface area modulation or salt film property modulations, for instance, its density, ionic conductivity, or thickness [41]. The size of both HF and LF loops increased appreciably with the inhibitor concentration, the magnitude of absolute impedance increased, and the phase angles shifted towards higher values. This could be ascribed to the formation of a film on the alloy surface [26].

**Figure 6:**
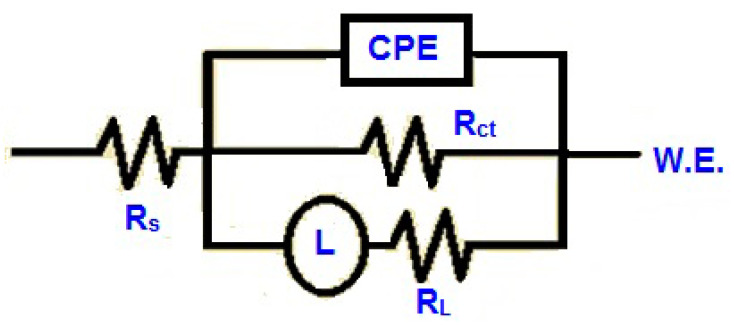
Equivalent electric circuits for quantitative estimation of EIS spectra.

To carry out an analysis of the EIS data, it was crucial to fit the data using an electric equivalent circuit (EEC). All plots were simulated using the most appropriate EEC, as shown in Figure 6. The EEC consisted of five elements, where *R*_s_ is the solution resistance, *R*_ct_ is the charge-transfer resistance, *CPE* is the constant phase element which corresponds to the double-layer capacitance (*C*), *L* is an inductive element, and *R*_L_ is the corresponding resistance. *CPE* was applied instead of actual capacitance, since the achieved plots had depressed semicircles. CPE is a combination of properties associated with both the surface and the electro-active species, and is independent of frequency. The application of the CPE is essential, owing to the distribution of relaxation times because of inhomogeneities present at the micro/nano level, like surface roughness/porosity, adsorption, and diffusion [42]. The depressed semicircles define the frequency dispersion during impedance analysis because of the inhomogeneity of the surface [37]. Polarization resistance (*R*_p_) was calculated as follows [9]:

(4) $$R_{p}=\frac{R_{t}\cdot R_{L}}{R_{t}+R_{L}}$$

The inhibition efficiency ($\eta_{\mathrm{imp}}$) was evaluated using the following equation:

(5) $$\eta_{\mathrm{imp}}\%=\frac{R_{p\left(i\right)}-R_{p\left(0\right)}}{R_{p\left(i\right)}}\;\times \;100$$

where $R_{p\left(0\right)}$ and $R_{p\left(i\right)}\;\;$ are polarization resistance values in the absence and presence of inhibitor, respectively.

The values of the impedance parameters are recorded in Table 3. The fitted data are in good agreement with the experimental data, as evidenced by their low *χ*^2^ values. Both *R*_ct_ and *R*_p_ values increased significantly with the addition of BMIm, indicating that the metal exhibited less corrosion in the presence of the inhibitor. The drop in CPE values with increasing inhibitor concentrations compared with the CPE values in blank solution may have been due to a reduction in local dielectric constant and/or increase in the thickness of the electrical double layer [43,44]. This suggests the adsorption of inhibitor molecules at the metal/solution interface [44]. $\eta_{\mathrm{imp}}$ increased with the inhibitor concentration and the maximum efficiency reached 98.4%, which again confirms that IL displays a good inhibitive effect on AA 6061 alloy in 1 M HCl. The inhibition efficiencies attained from mass loss ($\eta_{w}$ = 98.2%), potentiodynamic polarization tests ($\eta_{p}$ = 98%), and EIS ($\eta_{\mathrm{imp}}$ = 98.4%) were in good agreement.

### 3.5. SEM-EDX Analyses and Elemental Mapping

**Figure 7:**
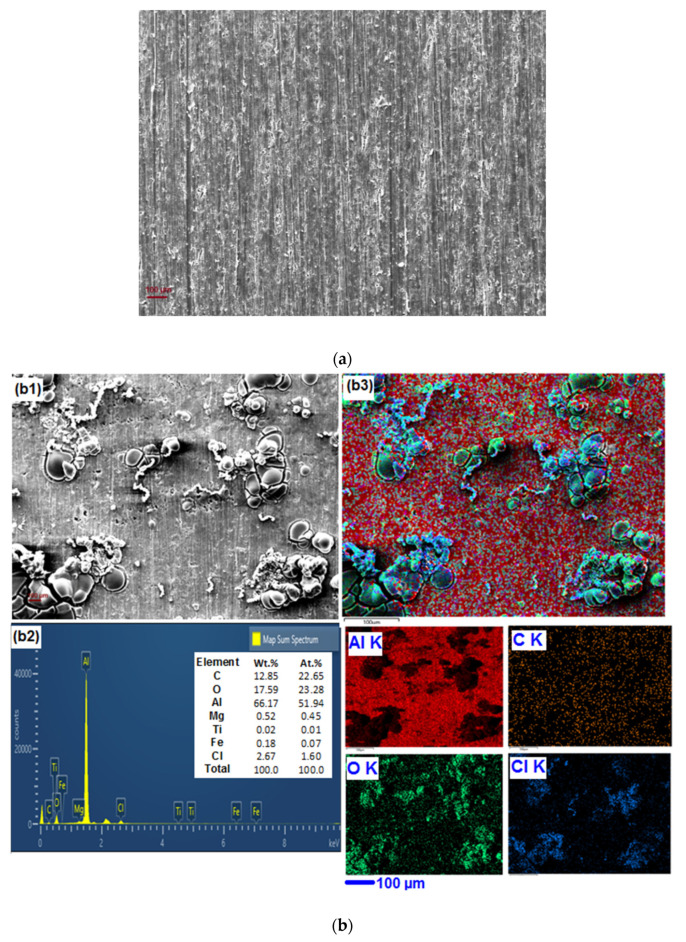

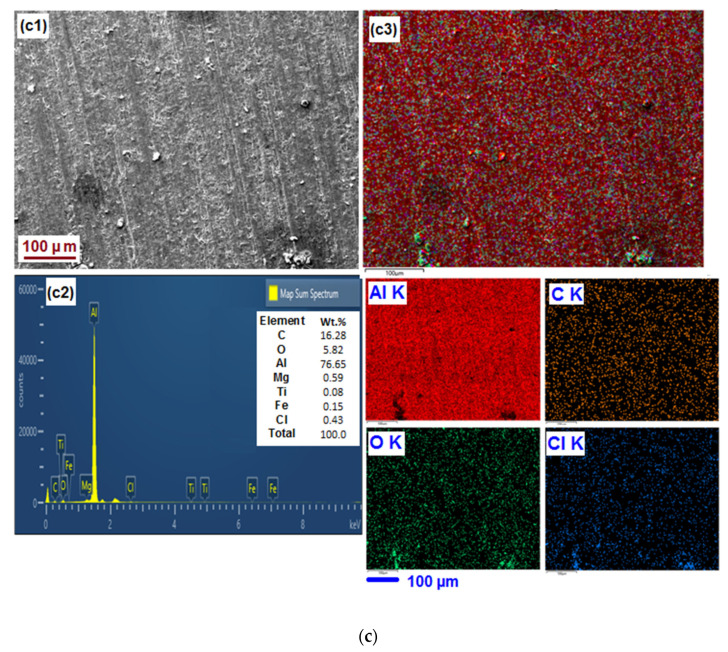
SEM micrograph and EDX images with their elemental mapping for different samples, (**a**) as polished; (**b1**–**b3**) after immersion in 1 M HCl in absence of inhibitor; (**c1**–**c3**) after immersion in 1 M HCl in presence of 3.0 mM inhibitor.

Topographic and elemental chemistry information of polished alloy surface before exposure, after exposure to acidic solution, and after exposure to an acidic solution in the presence of IL, was used to clarify the corrosion and inhibition mechanisms. Figure 7a shows SEM images of the polished AA 6061 alloy surface, and Figure 7b,c shows the SEM images and the corresponding EDX spectra of the AA 6061 alloy after immersion in 1 M HCl without and with 3 mM inhibitor for 90 min at 303 K. It may be noticed from Figure 7a that before immersion, the aluminum alloy samples appeared smooth, with polishing marks visible; however, after immersion in 1 M HCl solution without inhibitor, the surface showed the destructive attack of the corroding medium with damaged polishing marks, as is apparent in Figure 7b1. Moreover, the corrosion products seemed to be very uneven and the surface quite rough. Alternatively, Figure 7c1 demonstrates that there was less destruction on the surface of the alloy in the presence of inhibitor, and the alloy surface is smoother, which additionally confirms the inhibition ability. The EDX spectrum illustrates peaks which are analogous to the elements present in the alloy, along with their weighted proportions. Representative elemental mapping images are also shown, along with their corresponding SEM images, which provide qualitative support to the experimental results. The corresponding EDX spectra in Figure 7b2 show a higher amount of Cl (due to HCl), and more Cl accumulated at the corrosion sites (Figure 7b3) than appeared in the sample with inhibitor (Figure 7c1,c2). The observation that the amount of C increased in the samples dipped in the solution having BMIm further confirms the adsorption of inhibitor on the alloy surface. S and N were not observed in EDX, possibly due to very low adsorption or adsorption levels, i.e., below detection limits of EDX; this possibility was explored by XPS analysis.

### 3.6. Contact Angle Analysis

**Figure 8:**
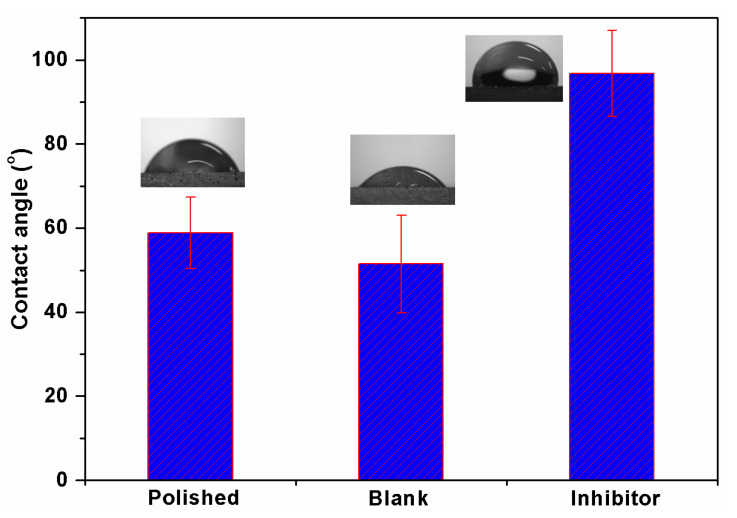
Variation of contact angle for different samples.

The surface hydrophilic character of the samples was studied by water contact angle measurements. The samples were immersed in 1 M HCl without and with 3.0 mM BMIm for 90 min before measuring the contact angle. The measured contact angle of water droplets on different samples are presented in Figure 8. The measurement was done at six different places and average values are reported. The contact angle for the polished sample was 58.8° ± 8. After immersion in 1 M HCl, the contact angle reduced and reached a value of 51.4° ± 11, which shows a more rough and hydrophilic surface, as also evidenced by the SEM analysis. It has been reported that a greater surface roughness results in an easier spread of water droplets on metal or alloy surfaces [45]. On the other hand, the surface after immersion in the solution with inhibitor became more hydrophobic; this was obvious from its greater contact angle value (96.8° ± 10). This evidence indicates that the surface was less prone to wetting by water in the presence of inhibitor, which is an important factor defining corrosion protection in the corrosive medium [46].

### 3.7. X-ray Photoelectron Spectroscopy (XPS) Analysis

**Figure 9:**
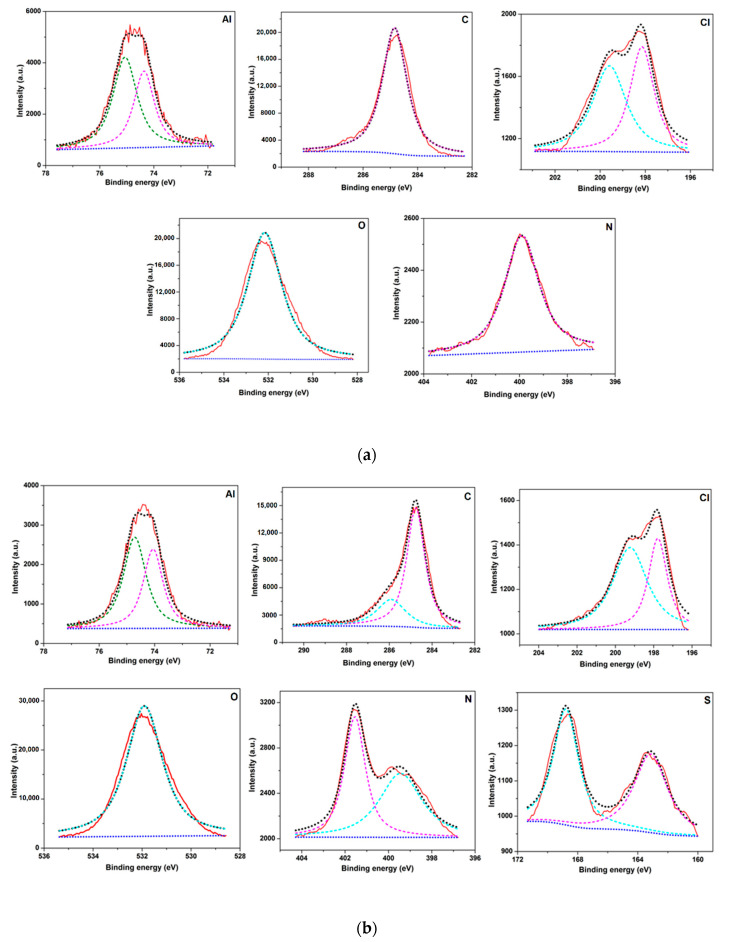
XPS peaks for passive films of the AA 6061 alloy exposed to 1 M HCl solution, (**a**) without inhibitor; (**b**) with 3.0 mM inhibitor.

High-resolution XPS spectra were measured in the binding energy range of C 1s, Al 2p, O 1s, N 1s, S 2p, and Cl 2p photopeaks to determine the interaction between the investigated IL and the Al alloy surface. The investigation was executed after exposure to 1 M HCl solution without and with 3 mM of IL. The purpose of the implemented analysis was a qualitative and quantitative comparison of several chemical species present at the metal/electrolyte interface and the determination of the inhibition mechanism. The results obtained by the XPS study are presented in Figure 9. The Al peak can be deconvoluted into two at about 74.3 eV and 75.1 eV, which are associated with the metallic and oxide peak of Al_2_O_3_ [47]. Only one peak of the C 1s spectrum was obtained for a sample immersed in solution without inhibitor, located at 284.7 eV, because of the adventitious adsorption of carbon on the alloy surface due to air exposure [48,49]. One extra peak is revealed for the sample immersed in the solution having BMIm at 285.9, which might have originated from the C–N bond of the adsorbed inhibitor [50]. XPS analyses of N 1s illustrate a strong indication of chemical interactions between the inhibitor and the surface of the metal, and established that the BMIm was adsorbed on the alloy surface. Only one characteristic peak at 399.1 eV is depicted for samples immersed in 1 M HCl without the inhibitor but deconvolution of the N1s signal may be fitted into two peaks in a solutions with inhibitor. The peak at 399.1 eV was attributed to the coordinated nitrogen atom or C–N-metal connection [51]. The peak at 401.5 eV was attributed to the coordinated nitrogen in the imidazolium ring with the AA 6061alloy surface [44]. The S 2p peaks were only observed for the samples immersed in solution having inhibitor at 163.1eV and 168.7 eV. These can be attributed to 2p_1/2_ and 2p_3/2_ covalent bonds between C and S [52]. The distinct appearances of nitrogen and sulfur peaks indicate the adsorption of BMIm on the alloy surface. The O peak was located at 532.1 eV and attributed to metal oxide species. Alternatively, the Cl 2p signal can be deconvoluted into two peaks for both samples at 198.1 and 199.5, which indicates that Cl^−^ anions had interacted with the substrate [53]. The atomic percentages of these signals are presented in Table 4.

## 4. Discussion

Analogous to other major organic corrosion inhibitors, ILs also inhibit metal corrosion by suppressing reactions at the anodic and/or cathodic sites at the surface of metal [54,55]. Hence, inhibition of metal corrosion in the presence of ILs comprises the blocking of anodic oxidative metal dissolution along with hydrogen evolution by cathodic reactions [55]. The mechanism of Al corrosion in HCl solution has been postulated [56] and the anodic dissolution of aluminum can be represented, according to [28], as Equations (6) and (7):

Al + Cl^−^ → AlCl_ads_^−^ (6)

AlCl_ads_^−^ + Cl^−^ → AlCl_2_^+^ + 3 e^−^ (7)

The cationic part of ionic liquid (BMIm^+^) can interact electrostatically with AlCl_ads_^−^ ions and then check the oxidation reaction of AlCl_ads_^−^ to AlCl_2_^+^ as revealed by Equation (7).

Firstly, hydronium ions adsorption and hydrogen gas evolution occur at cathodic sites at the same time. At the cathode, BMIm^+^ competes with hydrogen ions for electrons, leading to the formation of [Al-BMIm_ads_] [13,55,57].

(8) $${\mathrm{Al}}_{2}O_{3}\;+\;H_{3}O^{+}\;+\;e^{-}\;↔\;{\mathrm{Al}}_{2}O_{3}(H_{2}O)\;+\;\;\frac{1}{2}\;H_{2}$$

Al + BMIm^+^ + e^−^ → [Al-BMIm_ads_] (9)

**Figure 10:**
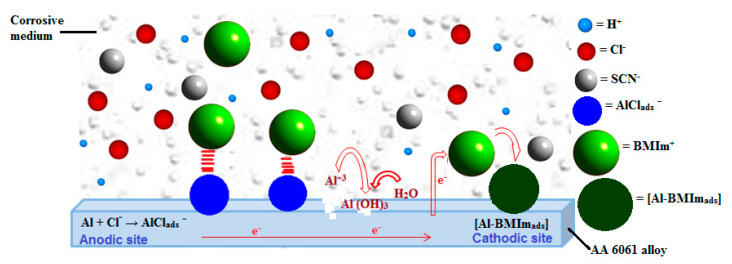
Mechanism of corrosion protection by BMIm IL in 1 M HCl medium.

BMIm^+^ has a big molecular size and thus substitutes a larger number of water molecules from the surface of the metal. After their adsorption, BMIm^+^ accepts electrons from the metal, which leads to the formation of electrically neutral IL molecules that also act as inhibitors. The neutral species transfer their nonbonding electrons which are present on heteroatoms, and π-electrons into the d-orbitals of the metal atoms of the surface, followed by the formation of coordinate bonds between metal and the adsorbed IL, as reported for numerous organic inhibitors [58,59,60]. Since metals are electron-rich already, this sort of donation results in interelectronic repulsion, which again leads to the transfer of electrons from the d-orbitals of the surface metallic atoms to antibonding molecular orbitals of the ILs, called retrodonation. Mutual donation and retrodonation reinforce each other through synergism, resulting in the blocking of the metal surface, which inhibits metal corrosion [14,59,61]. This mechanism is illustrated in Figure 10.

## 5. Conclusions

The corrosion inhibition behavior of imidazolium-based ionic liquid, BMIm, was studied for AA6061 alloy in 1 M HCl to explore its inhibitive ability. The obtained results led to the following conclusions:

- BMIm is an effective corrosion inhibitor for AA6061; the maximum inhibition efficiencies ($\eta_{w}$) were 98.2%, 86.6% and 41.2% at 303 K, 333 K, and 363 K, respectively, at 3.0 mM concentration. Inhibition efficiency increases with the inhibitor concentration, but decreases with the increase of temperature. $\eta_{w}$ increases with immersion time at 303 K from 30 to 90 min, and then decreases slightly but for other temperatures; it decreases regularly with an increase in immersion time. $\eta_{w}$ at 363 K was 71.4% if the samples had been preadsorbed in inhibitor and then tested.
- Polarization results show that BMIm can efficiently diminish corrosion of AA 6061 in HCl solution, and can be deemed as a mixed-type inhibitor with principal control on cathodic processes, thereby reducing the overall rate of corrosion. The EIS results indicate the development of a more protective passive film on the sample surface immersed in an acid solution containing inhibitor.
- Surface morphology analyses by SEM revealed less surface damage in the presence of inhibitor, verifying the effectiveness of the BMIm, while EDX and elemental mapping offers graphic and qualitative support to the results from experiments. A contact angle analysis suggested that the inhibited Al 6061 surface was hydrophobic in nature. XPS analysis established the formation of a protective film by the inhibitor on the AA6061 alloy surface.
- ILs have a promising future in the field of corrosion inhibition as green corrosion inhibitors for various alloys. Among the numerous existing ILs, imidazole-based ionic liquids have been most extensively used. More research needs to be carried out in other industrially relevant corrosive environments like CO_2_, H_2_S, and NaCl to determine the inhibitive behavior of IL-based compounds for various alloys in these environments.

## Figures and Tables

**Table 1:** Composition of AA6061 alloy (wt.%).

Fe	Si	Cu	Mn	Mg	Ti	Zn	Al
0.15	0.53	0.26	0.06	0.92	0.04	0.02	Bal.

**Table 2:** Polarization parameters for the corrosion of AA 6061 alloy in 1 M HCl containing various concentrations of inhibitor at 303 K.

Inhibitor Conc. (mM)	*E*_corr_ (V vs. SCE)	*I*_corr_ (µAcm^−2^)	*β*_c_ (mV dec^−1^)	*β*_a_ (mV dec^−1^)	Corrosion Rate (mmy^−1^)	Polarization Resistance (Ω)	*IE* (%)
0	−0.71	1030	80	139	12.06	4.9	--
1	−0.73	59.3	98	21	0.689	124.6	94.2
2	−0.77	29.5	77	40	0.271	504.3	97.1
3	−0.76	22.3	68	90	0.254	582.0	97.8
4	−0.79	20.7	60	52	0.223	665.6	98.0

**Table 3:** EIS data for the corrosion of AA 6061 alloy in 1 M HCl in the existence of different concentration of inhibitor at 303 K.

BMIm Conc. (mM)	*R*_s_ (Ω cm^2^)	*CPE* (μF cm^−2^)	*R*_ct_ (Ω cm^2^)	*R*_p_ (Ω cm^2^)	*C*_dl_ (μF cm^−2^)	*χ* ^2^	*η* (%)
Blank	17.8	9.3 × 10^−5^	39.1	18.74	4.7 × 10^−5^	0.0035	-
1.0	15.2	3.9 × 10^−5^	3.7 × 10^2^	317.7	2.8 × 10^−5^	0.0022	94.0
2.0	23.6	2.0 × 10^−5^	7.8 × 10^2^	672.1	1.7 × 10^−5^	0.0029	97.2
3.0	27.3	2.2 × 10^−5^	1.2 × 10^3^	1032.3	1.6 × 10^−5^	0.0011	98.2
4.0	27.2	1.3 × 10^−5^	3.8 × 10^3^	2756.9	9.1 × 10^−6^	0.0032	98.4

**Table 4:** Compositions of surface elements (at.%) from the XPS investigation for AA 6061alloy samples immersed in 1 M HCl without and with inhibitor.

Element	1 M HCl	1 M HCl + 3 mM IL
Al	12.3	13.6
C	52.6	41.6
Cl	1.6	0.98
O	31.8	39.3
N	1.2	03.3
S	0.4	1.2

## References

[1] Wang X.H., Zhong S.Y., Song Y.H., Liu H., Huang A.L., Hu Q.G., Wang G.R., Chai S.S. (2020). Effect of tantalum film on corrosion behaviour of AA6061 aluminium alloy in hydrochloric acid- and chloride-containing solutions. Trans. IMF.

[2] Goswami R., Lynch S., Holroyd N.J.H., Knight S.P., Holtz R.L. (2013). Evolution of Grain Boundary Precipitates in Al 7075 Upon Aging and Correlation with Stress Corrosion Cracking Behavior. Metall. Mater. Trans. A.

[3] Khaled K.F., Al-Qahtani M.M. (2009). The inhibitive effect of some tetrazole derivatives towards Al corrosion in acid solution: Chemical electrochemical and theoretical studies. Mater. Chem. Phys..

[4] Xhanariab K., Finšgar M. (2016). Organic corrosion inhibitors for aluminium and its alloys in acid solutions: A review. RSC Adv..

[5] Padash R., Rahimi-Nasrabadi M., Rad A.S., Sobhani-Nasab A., Jesionowski T., Ehrlich H. (2019). A theoretical study of two novel Schiff bases as inhibitors of carbon steel corrosion in acidic medium. Appl. Phys. A.

[6] He X.K., Jiang Y.M., Li C., Wang W.C., Hou B.L., Wu L.Y. (2014). Inhibition properties and adsorption behavior of imidazoleand 2-phenyl-2-imidazoline on AA5052 in 1.0 M HCl solution. Corros. Sci..

[7] Rodič P., Milošev I., Lekka M., Andreatta F., Fedrizzi L. (2018). Corrosion behaviour and chemical stability of transparent hybrid sol-gel coatings deposited on aluminium in acidic and alkaline solutions. Prog. Org. Coat..

[8] Deng S.D., Li X.H. (2012). Inhibition by JasminumnudiflorumLindl leaves extract of the corrosion of aluminum in HCl solution. Corros. Sci..

[9] Fares M.M., Maayta A.K., Al-Qudah M.M. (2012). Pectin as promising green corrosion inhibitor of aluminum in hydrochloric acid solution. Corros. Sci..

[10] Oguzie E.E. (2007). Corrosion inhibition of aluminum in acidic and alkaline media by Sansevieriatrifasciata extract. Corros. Sci..

[11] Palou R.M., Olivares-Xomelt O., Likhanova N.V. (2014). Environmentally Friendly Corrosion Inhibitors. Development in Corrosion Protection.

[12] Kowsari E., Arman S.Y., Shahini M.H., Zandi H., Ehsani A., Naderi R., Hanza A.P., Mehdipour M. (2016). In situ synthesis, electrochemical and quantum chemical analysis of an amino acid-derived ionic liquid inhibitor for corrosion protection of mild steel in 1M HCl solution. Corros. Sci..

[13] Shetty S.K., Shetty A.N. (2017). Eco-friendly benzimidazolium based ionic liquid as a corrosion inhibitor for aluminum alloy composite in acidic media. J. Mol. Liq..

[14] Uerdingen M., Treber C., Balser M., Schmitt G., Werner C. (2005). Corrosion behavior of ionic liquids. Green Chem..

[15] Wang B., Qin L., Mu T., Xue Z., Gao G. (2017). Are ionic liquids chemically stable?. Chem. Rev..

[16] Xue Z.M., Zhang Y.W., Zhou X.Q., Cao Y.Y., Mu T.C. (2014). Thermal stabilities and decomposition mechanism of amino-and hydroxyl-functionalized ionic liquids. Thermochim. Acta.

[17] Blanchard L.A., Hancu D., Beckman E.J., Brennecke J.F. (1999). Green processing using ionic liquids and CO_2_. Nature.

[18] Efimova A., Varga J., Matuschek G., Saraji-Bozorgzad M.R., Denner T., Zimmermann R., Schmidt P. (2018). Thermal Resilience of Imidazolium-Based Ionic Liquids—Studies on Short- and Long-Term Thermal Stability and Decomposition Mechanism of 1-Alkyl-3-methylimidazolium Halides by Thermal Analysis and Single-Photon Ionization Time-of-Flight Mass Spectrometry. J. Phys. Chem. B.

[19] Qiang Y.J., Zhang S.T., Guo L., Zheng X.W., Xiang B., Chen S.J. (2017). Experimental and theoretical studies of four allylimidazolium-based ionic liquids as green inhibitors for copper corrosion in sulfuric acid. Corros. Sci..

[20] Trombetta F., de Souza R.F., de Souza M.O., Borges C.B., Panno N.F., Agostini Martini E.M. (2011). Stability of aluminium in 1-butyl-3-methylimidazolium tetrafluoroborate ionic liquid and ethylene glycol mixtures. Corros. Sci..

[21] Wu Q.L., Zhang Z.H., Dong X.M., Yang J.Q. (2013). Corrosion behavior of low-alloy steel containing 1% chromium in CO_2_ environments. Corros. Sci..

[22] Bousskri A., Anejjar A., Messali M., Salghi R., Benali O., Karzazi Y., Jodeh S., Zougagh M., Ebenso E.E., Hammouti B. (2015). Corrosion inhibition of carbon steel in aggressive acidic media with 1-(2-(4-chlorophenyl)-2-oxoethyl)pyridaziniumbromide. J. Mol. Liq..

[23] Ibrahim M.A.M., Messali M., Moussa Z., Alzahrani A.Y., Alamry S.N., Hammouti B. (2011). Corrosion inhibition of carbon steel by imidazolium and pyridiniumcations ionic liquids in acidic environment. Port. Electrochim. Acta.

[24] Sherif E.M., Abdo H.S., Abedin S.Z.E. (2015). Corrosion inhibition of cast iron in Arabian gulf seawater by two different ionic liquids. Materials.

[25] Luna M.C., Manh T.L., Sierra R.C., Flores J.V.M., Rojas L.L., Estrada E.M.A. (2019). Study of corrosion behavior of API 5L X52 steel in sulfuric acid in the presence of ionic liquid 1-ethyl 3-methylimidazolium thiocyanate as corrosion inhibitor. J. Mol. Liq..

[26] Li X.H., Deng S.D., Fu H. (2011). Inhibition by tetradecylpyridinium bromide of the corrosion of aluminium in hydrochloric acid solution. Corros. Sci..

[27] Zhao T.P., Mu G.N. (1999). The adsorption and corrosion inhibition of anion surfactants on aluminium surface in hydrochloric acid. Corros. Sci..

[28] Li X.H., Deng S.D., Xie X.G. (2014). Experimental and theoretical study on corrosion inhibition of o-phenanthroline for aluminum in HCl solution. J. Taiwan Inst. Chem. E.

[29] Ashassi-Sorkhabi H., Shabani B., Aligholipour B., Seifzadeh D. (2006). The effect of some Schiff bases on the corrosion of aluminum in hydrochloric acid solution. Appl. Surf. Sci..

[30] Cao C.N. (2004). Corrosion Electrochemistry Mechanism.

[31] Mousavi M., Mohammadalizadeh M., Khosravan A. (2011). Theoretical investigation of corrosion inhibition effect of imidazole and its derivatives on mild steel using cluster model. Corros. Sci..

[32] Abdallah M., Sobhi M., Altass H.M. (2016). Corrosion inhibition of aluminum in hydrochloric acid by pyrazinamide derivatives. J. Mol. Liq..

[33] Arukalam I.O., Madu I.O., Ijomah N.T., Ewulonu C.M., Onyeagoro G.N. (2014). Acid corrosion inhibition and adsorption behavior of ethyl hydroxyethyl cellulose on mild steel corrosion. J. Mater..

[34] Pinto G.M., Nayak J., Shetty A.N. (2011). Corrosion inhibition of 6061 Al–15 vol. pct. SiC(p) composite and its base alloy in a mixture of sulfuric acid and hydrochloric acid by4-(N,N-dimethylamino) benzaldehyde thiosemicarbazone. Mater. Chem. Phys..

[35] Yurt A., Ulutas S., Dal H. (2006). Electrochemical and theoretical investigation on the corrosion of aluminium in acidic solution containing some Schiff bases. Appl. Surf. Sci..

[36] Bessone J., Mayer C., Jutter K., Lorenz W. (1983). AC-impedance measurements on aluminium barrier type oxide films. Electrochim. Acta.

[37] Brett C.M.A. (1992). On the electrochemical behaviour of aluminium in acidic chloride solution. Corros. Sci..

[38] MetikoŠ-Hukovi C.M., Babi C.R., Gruba C.Z. (1998). Corrosion protection of aluminum in acidic chloride solutions with nontoxic inhibitors. J. Appl. Electrochem..

[39] Abd El Rehim S.S., Hassan H.H., Amin M.A. (2001). Corrosion inhibition of aluminum by 1,1(lauryl amido) propyl ammonium chloride in HCl solution. Mat. Chem. Phys..

[40] Noor E.A. (2009). Evaluation of inhibitive action of some quaternary N-heterocyclic compounds on the corrosion of Al–Cu alloy in hydrochloric acid. Mat. Chem. Phys..

[41] Bessone J.B., Salinas D.R., Mayer C.E., Ebert M., Lorenz W.J. (1992). An EIS study of aluminium barrier-type oxide films formed in different media. Electrochim. Acta.

[42] Flores E.N., Chong Z., Omanovic S. (2005). Characterization of Ni, NiMo, NiW and NiFe electroactive coatings as electrocatalysts for hydrogen evolution in an acidic medium. J. Mol. Catal. A Chem..

[43] McCafferty E., Hackerman N. (1972). Double Layer Capacitance of Iron and Corrosion Inhibition with Polymethylene Diamines. J. Electrochem. Soc..

[44] Lagrenee M., Mernari B., Bouanis M., Traisnel M., Bentiss F. (2002). Study of the mechanism and inhibiting efficiency of 3,5-bis(4-metylthiophenyl)-4H-1,2,4-triazole on mild steel corrosion in acidic media. Corros. Sci..

[45] Ramezanzadeh B., Akbarian M., Karati M.R., Mahdavian M., Alibakhshi E., Kardar P. (2017). Corrosion protection of steel with zinc phosphate conversion coating andpost-treatment by hybrid organic-inorganic sol-gel based silane film. J. Electrochem. Soc..

[46] Antonijevic M.M., Milić S.M., Petrović M.B. (2009). Films formed on copper surface in chloride media in the presence of azoles. Corros. Sci..

[47] Tamilarasan R., Sreekanth A. (2013). Spectroscopic and DFT investigations on the corrosion inhibition behavior of tris (5-methyl-2-thioxo-1,3,4-thiadiazole) borate on high carbon steel and aluminium in HCl media. RSC Adv..

[48] Piao H., McIntyre N.S. (2002). Adventitious carbon growth on aluminium and gold-aluminium alloy surfaces. Surf. Interface Anal..

[49] Barr T.L., Seal S. (1995). Nature of the use of adventitious carbon as a binding energy standard. J. Vac. Sci. Technol. Vac. Surf. Film..

[50] Ryl J., Wysocka J., Cieslik M., Gerengi H., Ossowski T., Krakowiak S., Niedzialkowski P. (2019). Understanding the origin of high corrosion inhibition efficiency of beeproducts towards aluminium alloys in alkaline environments. Electrochim. Acta.

[51] Zarrok H., Zarrouk A., Hammouti B., Salghi R.C., Bentiss J.M. (2012). Corrosion control of carbon steel in phosphoric acid by purpald—Weight loss, electrochemical and XPS studies. Corros. Sci..

[52] Tourabi M., Nohair K., Traisnel M., Jama C., Bentiss F. (2013). Electrochemical and XPS studies of the corrosion inhibition of carbon steel in hydrochloric acid pickling solutions by 3,5-bis(2-thienylmethyl)-4-amino-1,2,4-triazole. Corros. Sci..

[53] Arellanes-Lozada P., Olivares-Xometl O., Likhanova N.V., Lijanova I.V., Vargas-García J.R., Hernández-Ramírez R.E. (2018). Adsorption and performance of ammonium-based ionic liquids as corrosion inhibitors of steel. J. Mol. Liq..

[54] Likhanova N.V., Domínguez-Aguilar M.A., Olivares-Xometl O., Nava-Entzana N., Arce E., Dorantes H. (2010). The effect of ionic liquids with imidazolium and pyridiniumcations on the corrosion inhibition of mild steel in acidic environment. Corros. Sci..

[55] Zhang D.S., Li L.D., Cao L.X., Yang N.F., Huang C.B. (2001). Studies of corrosion inhibitors for zinc–manganese batteries: Quinoline quaternary ammonium phenolates. Corros. Sci..

[56] Bereket G., Pinarbsi A. (2004). Electrochemical thermodynamic and kinetic studies of the behaviour of aluminium in hydrochloric acid containing various benzotriazole derivatives. Corros. Eng. Sci. Technol..

[57] Antonijevic M.M., Petrovic M.B. (2008). Copper corrosion inhibitors. Int. J. Electrochem. Sci..

[58] Fuchs-Godec R. (2006). The adsorption, CMC determination and corrosion inhibition of some N-alkyl quaternary ammonium salts on carbon steel surface in 2M H_2_SO_4_. Colloid Surf. A.

[59] Verma C., Ebenso E.E., Vishal Y., Quraishi M.A. (2016). Dendrimers: A new class of corrosion inhibitors for mild steel in 1M HCl: Experimental and quantum chemical studies. J. Mol. Liq..

[60] Haque J., Srivastava V., Verma C., Quraishi M.A. (2017). Propionic Acid as New and Green Corrosion Inhibitor for Mild Steel in 1 M Hydrochloric Acid Solution. J. Mol. Liq..

[61] Thirumalairaj B., Jaganathan M. (2016). Corrosion protection of mild steel by a new binary inhibitor system in hydrochloric acid solution. Egypt. J. Pet..

